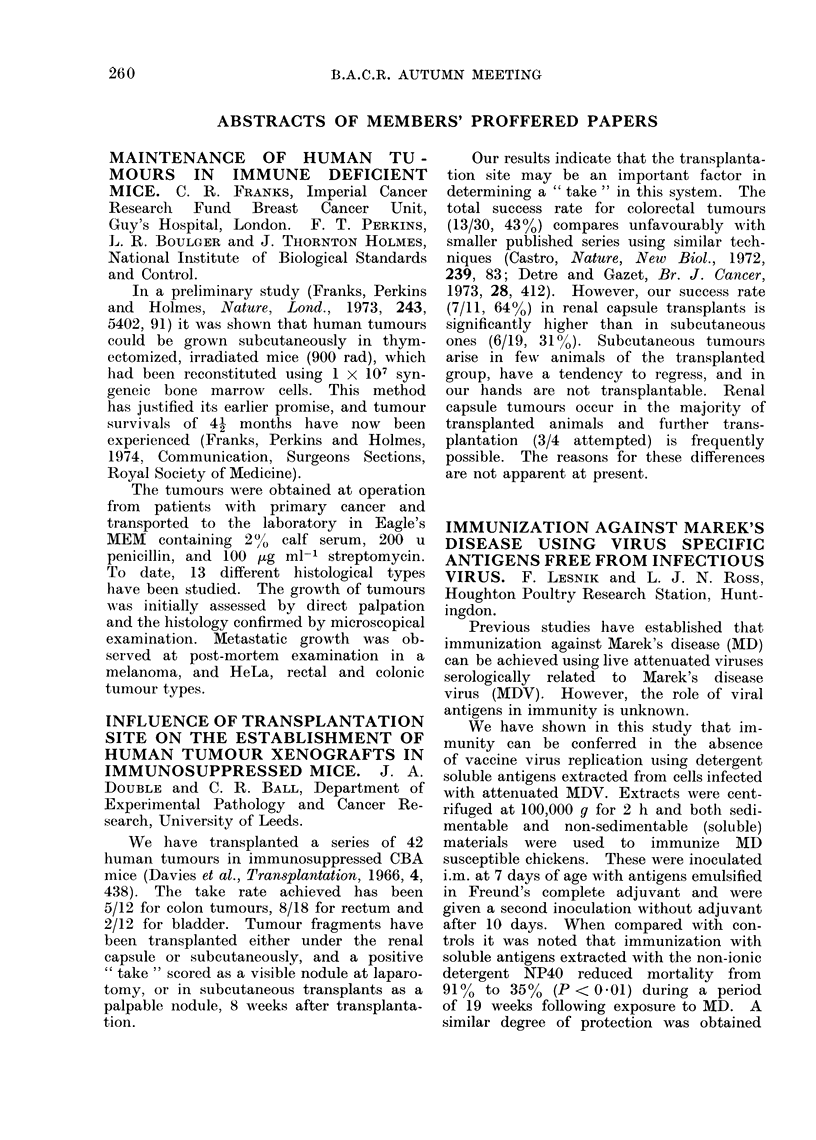# Proceedings: Influence of transplantation site on the establishment of human tumour xenografts in immunosuppressed mice.

**DOI:** 10.1038/bjc.1975.37

**Published:** 1975-02

**Authors:** J. A. Double, C. R. Ball


**INFLUENCE OF TRANSPLANTATION SITE ON THE ESTABLISHMENT OF HUMAN TUMOUR XENOGRAFTS IN IMMUNOSUPPRESSED MICE.** J. A. DOUBLE and C. R. BALL, Department of Experimental Pathology and Cancer Research, University of Leeds.

We have transplanted a series of 42 human tumours in immunosuppressed CBA mice (Davies *et al.*, *Transplantation*, 1966, **4**, 438). The take rate achieved has been 5/12 for colon tumours, 8/18 for rectum and 2/12 for bladder. Tumour fragments have been transplanted either under the renal capsule or subcutaneously, and a positive " take " scored as a visible nodule at laparotomy, or in subcutaneous transplants as a palpable nodule, 8 weeks after transplantation.

Our results indicate that the transplantation site may be an important factor in determining a " take " in this system. The total success rate for colorectal tumours (13/30, 43%) compares unfavourably with smaller published series using similar techniques (Castro, *Nature, New Biol.*, 1972, **239**, 83; Detre and Gazet, *Br. J. Cancer*, 1973, **28**, 412). However, our success rate (7/11, 64%) in renal capsule transplants is significantly higher than in subcutaneous ones (6/19, 31%). Subcutaneous tumours arise in few animals of the transplanted group, have a tendency to regress, and in our hands are not transplantable. Renal capsule tumours occur in the majority of transplanted animals and further transplantation (3/4 attempted) is frequently possible. The reasons for these differences are not apparent at present.